# Do intravascular hypovolaemia and hypervolaemia result in changes in pulmonary blood volume?

**DOI:** 10.1186/cc14252

**Published:** 2015-03-16

**Authors:** JJ Vos, TW Scheeren, SA Loer, A Hoeft, JK Wietasch

**Affiliations:** 1 University of Groningen, University Medical Center Groningen, the Netherlands; 2 Institute for Cardiovascular Research, VU University Medical Centre, Amsterdam, the Netherlands; 3 University of Bonn, Germany

## Introduction

Hypovolaemia is generally believed to induce centrali sation of blood volume. Therefore, we evaluated whether hypovolaemia and hypervolaemia result in a change in central blood volume (that is, pulmonary blood volume (PBV)) and we explored the effects on the distribution between PBV and circulating blood volume (Vd circ).

## Methods

After local District Governmental Animal Investigation Committee approval, blood volume was altered in both directions randomly in steps of 150 ml (mild) to 450 ml (moderate) either by haemorrhage, retransfusion of blood, or infusion of colloids in six Foxhound dogs. The anaesthetised dogs were allowed to breathe spontaneously. Blood volumes were measured using the dye dilution technique: PBV was measured as the volume of blood between the pulmonary and aortic valve, and Vd circ by two-compartmental curve fitting [1,2]. The PBV/Vd circ ratio was used as a measure of blood volume distribution. A linear mixed model was used for analysing the influence of blood volume alterations on the measured haemodynamic variables and blood volumes.

## Results

**Figure 1:**
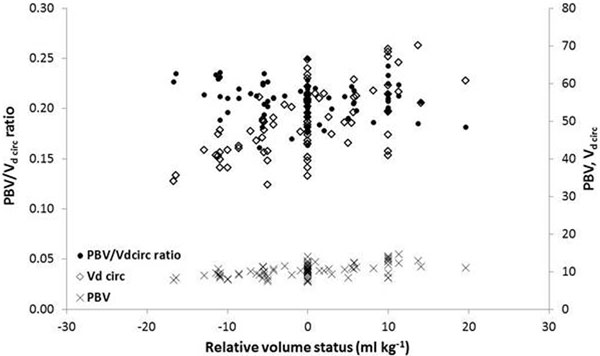


A total of 68 alterations in blood volume resulted in changes in Vd circ ranging from -33 to +31% (Figure 1). PBV decreased during mild and moderate haemorrhage, while during retransfusion PBV increased during moderate hypervolaemia only. The PBV/Vd circ ratio remained constant during all stages of hypovolaemia and hypervolaemia (Figure 1).

## Conclusion

Mild to moderate alterations of blood volume result in changes of PBV and Vd circ. However, against the traditional belief of centralisation we could show that the cardiovascular system preserves the distribution of blood between central and circulating blood volume in anaesthetised dogs.
